# Chronic Lung Injury by Constitutive Expression of Activation-Induced Cytidine Deaminase Leads to Focal Mucous Cell Metaplasia and Cancer

**DOI:** 10.1371/journal.pone.0117986

**Published:** 2015-02-06

**Authors:** Jiro Kitamura, Munehiro Uemura, Mafumi Kurozumi, Makoto Sonobe, Toshiaki Manabe, Hiroshi Hiai, Hiroshi Date, Kazuo Kinoshita

**Affiliations:** 1 Department of Thoracic Surgery, Faculty of Medicine, Kyoto University, Kyoto, Japan; 2 Department of Thoracic Surgery, Nagahama City Hospital, Nagahama, Japan; 3 Shiga Medical Center Research Institute, Moriyama, Japan; 4 Graduate School of Medicine, Kyoto University, Kyoto, Japan; Institut Pasteur, FRANCE

## Abstract

Activation-induced cytidine deaminase (AID) is an enzyme required for antibody diversification, and it causes DNA mutations and strand breaks. Constitutive AID expression in mice invariably caused lung lesions morphologically similar to human atypical adenomatous hyperplasia (AAH), which can be a precursor of bronchioloalveolar carcinoma. Similar to AAH, mouse AAH-like lesion (MALL) exhibited signs of alveolar differentiation, judging from the expression of alveolar type II (AT2) cell marker surfactant protein C (SP-C). However, electron microscopy indicated that MALL, which possessed certain features of a mucous cell, is distinct from an AAH or AT2 cell. Although MALL developed in all individuals within 30 weeks after birth, lung tumors occurred in only 10%; this suggests that the vast majority of MALLs fail to grow into visible tumors. MALL expressed several recently described markers of lung alveolar regeneration such as p63, keratin 5, keratin 14, leucine-rich repeat containing G protein-coupled receptor 5 (Lgr5), and Lgr6. Increased cell death was observed in the lungs of AID transgenic mice compared with wild-type mice. Based on these observations, we speculate that MALL is a regenerating tissue compensating for cellular loss caused by AID cytotoxicity. AID expression in such regenerating tissue should predispose cells to malignant transformation via its mutagenic activity.

## Introduction

Lung cancer is the leading cause of cancer deaths worldwide [1,2], and smoking accounts for approximately 80% of lung cancer cases [3]. On the other hand, lung diseases such as chronic obstructive pulmonary disease, infectious pneumonia, idiopathic interstitial pneumonia, and tuberculosis—all of which cause inflammation in lung tissue—increase the risk of lung cancer independent of tobacco use [4]. With the decline in smoking, the prevention of tobacco-independent lung cancer has become relatively important [2,3]. The biggest question to be answered is how oncogenic mutations occur in tobacco-independent lung cancer.

Recent studies suggest the involvement of cytidine deaminases in the development of cancers of the gastrointestinal tract, mammary gland, and prostate [5–9]. Activation-induced cytidine deaminase (AID), a member of the cytidine deaminase family, is an essential enzyme for somatic hypermutation and class-switch recombination of antibody genes. We previously reported that AID was expressed in several types of gastrointestinal and hepatobiliary cancers that occur in the background of chronic inflammation [5–7]. Transgenic expression of AID in mice causes various types of tumors, including those of the lung, liver, and stomach and leukemia [10,11]. AID expression was also reported in human lung adenocarcinoma [12]. These observations suggest the mutagenic role of AID in inflammation-associated cancer.

Approximately 10% of AID transgenic mice develop macroscopic lung tumor within 90 weeks after birth [11]. However, all individuals (including those without lung tumors) possess microscopic lung lesions morphologically similar to human atypical adenomatous hyperplasia (AAH), a precursor of bronchioloalveolar carcinoma [10,13]. We initially speculated that this mouse AAH-like lesion (MALL) is a neoplastic lesion that eventually develops into adenocarcinoma in AID transgenic mice. We began to analyze MALL hoping to obtain an insight into the mechanism of AID-induced lung tumor in mice and inflammation-associated lung cancer in humans. However, our data suggested that MALL is not a neoplastic lesion but a transient structure expressing recently described markers of lung alveolar regeneration. In this study, we explore the causes and characteristics of MALL, describe how AID causes MALL and lung tumor in mice, and address its implications regarding human lung carcinogenesis.

## Materials and Methods

### Mice

The use of conditional transgenic C57BL/6 mice possessing a single copy of transgene containing CAG promoter-driven floxed green fluorescent protein (GFP) coding sequence followed by mouse AID coding sequence (AID cTg) was previously described [14]. By crossing this mouse once with a Cre transgenic mouse driven by tissue non-specific alkaline phosphatase (TNAP) promoter (TNAP-Cre mice with mixed background of C57BL/6 and 129/Sv after three backcrosses with C57BL/6 [15]), mice with germline deletion of GFP segment—and, thus, constitutive AID expression (AIDon)—were obtained. AIDon mice of C57BL/6 background with minor contribution of 129/Sv, diluted by backcrossing more than six times, were used. C57BL/6J mice were purchased from Japan SLC, Inc. (Shizuoka, Japan). All mice were fed ad libitum and were sacrificed by cervical dislocation for censoring, or observed immediately after spontaneous death. All animal experiments were approved by the Ethical Committee for Animal Experiments and performed as per the Guidelines for Animal Experiments of Shiga Medical Center.

### Gene mutation analysis

Lung tissue was dissected, embedded in OCT compound (Sakura Fineteck Japan Co., Ltd., Tokyo, Japan), and frozen immediately over liquid nitrogen. Then, 10-μm-thick sections were stained with hematoxylin and eosin (HE). Individual MALL was isolated by laser microdissection using LMD 6000 (Leica Microsystems GmbH, Wetzlar, Germany). Genomic DNA was isolated from each MALL using the QIAamp DNA Micro kit (Qiagen GmbH, Hilden, Germany). The *Trp53* exon 5–8, *Kras* exon 2, and *Egfr* exon 19–21 were amplified using a nested polymerase chain reaction (PCR) with the following primers: *Trp53*; forward 5′ GCG CCA TGG CCA TCT ACA A 3′, reverse 5′ TCT TCT GTA CGG CGG TCT CT 3′, nested forward 5′ TGG CCA TCT ACA AGA AGT CAC 3′, and nested reverse 5′ CAG GGC AGG CAC AAA CA 3′. *Kras*; forward 5′ CGG GTG TGT CCA CAG GGT ATA GCG T 3′, reverse 5′ AGT TGA GTT CTA GGC CGG TCA 3′, nested forward 5′ GTG TGT CCA CAG GGT ATA GCG TAC T 3′, and nested reverse 5′ GTT GAG TTC TAG GCC GGT CAG G, 3′. *Egfr* exon 19; forward 5′ CCC TTT CTG CCT TAG CAT GTC 3′, reverse 5′ GGC ACA TCC CTC AGT CAC TTA ACA C 3′, nested forward 5′ CTC TTG GAT TTG TAA TGT AGA GCC 3′, and nested reverse 5′ ATT GGG TGT AAT TTA TTA GTG CAT C 3′. *Egfr* exon 20; forward 5′ GAA GAT AGT GGC ATT TCC TAA ACT C 3′, reverse 5′ GCA TGA ACA CCA GGC TCG ATG 3′, nested forward 5′ ACA TCC CAC AGT GTA TTA GCA TTT A 3′, and nested reverse 5′ CTG CTT TTC TTA GTG CTC TTA CCA 3′. *Egfr* exon 21; forward 5′ GCC CAA GGA ACG TGA CGA AAG 3′, 5′ AGG GTG CTA CTG AAT CCG TGG TCA T 3′, nested forward 5′ TTG TCA TTC ATG CCA GAT AAT TCC A 3′, and nested reverse 5′ TTA CTA CTC CCA CCG AAA TCC A 3′. PCR products were purified with an exonuclease/phosphatase mixture (ExoSAP-IT, USB Corporation/Affymetrix, Inc., Cleveland, OH, USA) and sequenced using the nested PCR primers and the Big Dye Terminator v1.1 Cycle Sequencing Kit (Applied Biosystems Inc., Foster City, CA, USA). Sequencing products were treated with the Big Dye XTerminator Purification Kit (Applied Biosystems) and analyzed using the ABI 3130xl Genetic Analyzer (Applied Biosystems).

### Histology and immunohistochemistry

Tissue samples from mice were fixed in 10% (w/v) formaldehyde, embedded in paraffin, and stained with HE for histological examination. For immunohistochemistry, tissue samples were fixed via HOPE Fixative (Polysciences, Inc., Warrington, PA, USA). The specimens were embedded in paraffin. Immunohistochemistry was performed using avidin-biotin-peroxidase complex (ABC). Monoclonal anti-AID antibody (MAID-2) was described [16]. Other antibodies were purchased: keratin 5 (sc-17090; Santa Cruz Biotechnology, Inc., Santa Cruz, CA, USA), ΔN p63 (sc-8609; Santa Cruz Biotechnology), Clara cell 10kd protein (CC-10, sc-9772; Santa Cruz Biotechnology), epithelial cadherin (E-cadherin, 24E10; Cell Signaling, Danvers, MA, USA), leucine-rich repeat containing G protein-coupled receptor 5 (Lgr5, ab75732; Abcam, Cambridge, MA, USA), surfactant protein C (SP-C, sc-13976; Santa Cruz Biotechnology), Lgr6 (sc-99123; Santa Cruz Biotechnology), keratin 14 (PRB-155P; Covance, Berkeley, CA, USA), and podoplanin (53–5381; eBioscience, San Diego, CA, USA). Each section was counterstained with hematoxylin.

### Estimation of the number of MALLs in the whole lung

Total number of MALLs was estimated as follows: Density of MALL on section (N, per cm^2^) was calculated as a mean value for 11 sections of 4-μm thickness (T = 0.0004 cm) collected at 100-μm intervals. The number and area of MALL and the area of alveolar region in each section were measured on whole section images using ScanScope scanner and ImageScope software (Aperio Technologies, Inc., Vista, CA, USA). Mean diameter of MALL (D, cm) was estimated as 0.005 cm. A hypothetical cubic tissue mass with a side of 1 cm can be sliced into 1/T sections. Therefore, the total number of MALL counts in all 1/T sections will be N/T. A single MALL is sliced into D/T pieces and counted D/T times. Dividing total MALL count (N/T) with this redundancy factor (D/T), three-dimensional density of MALL can be expressed as N/D per cm^3^. Using the following values (N = 4.3, D = 0.005, bilateral lung volume of 1.0 cm^3^) for a 37-week-old mouse, the total number of MALL in the lungs is calculated as N/D × lung volume = 4.3/0.005 × 1.0 = 860.

### Electron microscopy

For electron microscopic analyses, AIDon mice were perfused from the right ventricle with phosphate-buffered saline (PBS) to remove blood. Their lungs were then perfusion-fixed with a mixture of 1.4% paraformaldehyde and 1% glutaraldehyde in PBS, and then cut into 2-mm cubes for transmission electron microscopy and immersion-fixed in the same agent used for perfusion fixation at 4°C overnight. The sections were then post-fixed in 1% osmium tetroxide in 0.1 M phosphate buffer for 1.5 h. Samples were then dehydrated in a graded ethanol series, cleared with propylene oxide, and embedded in Epon. Blocks were at first cut as semithin (1 μm) sections and stained with toluidine blue to identify MALL. Ultrathin (60–90 nm) sections were stained with uranyl acetate and lead citrate and examined using a transmission electron microscope, H7650 (Hitachi, Ltd., Tokyo, Japan).

### Apoptosis assay

Terminal deoxynucleotidyl transferase (TdT)-mediated dUTP nick endlabeling (TUNEL) was carried out to detect apoptotic DNA breaks in the lung and liver tissues using the TACS 2 TdT-DAB In Situ Apoptosis Detection Kit (Trevigen Inc., Gaithersburg, MD, USA). The slides were counterstained with 1% methyl green. To measure cell death in the lung and liver, TUNEL-positive and -negative alveolar wall cells and hepatocytes were counted until reaching a total number of 1,000 cells in 9–24 nonoverlapping, randomly chosen ×400 fields. Cells were counted using image analysis software from VH Analyzer (Keyence, Osaka, Japan). Data were collected from three individual mice.

### Proliferation assay

AIDon mice received a daily intraperitoneal injection of 1-mg 5-ethynyl-2'-deoxyuridine (EdU) in 0.4-ml PBS for 7 days. The mice were then studied at 1 and 20 days after the last injection. Cell proliferation in the 10-μm-thick frozen sections of lungs was detected using the Click-iT EdU Alexa Fluor 488 Imaging Kit (Molecular Probes, Invitrogen, Eugene, OR, USA). The sections were fixed in 3.7% (v/v) formaldehyde for 10 min. Next the tissues were permeabilized with 0.5% Triton X-100 in PBS at room temperature for 20 min. The Click-iT EdU reaction cocktail including Alexa Fluor 488 azide was prepared according to manufacturer’s instructions and added to cover the slide and incubated at room temperature in the dark for 30 min. Nuclei were visualized with the Hoechst 33342 stain. Slides were examined under a DM 5000B fluorescence microscope (Leica Microsystems). EdU positive cells in the lung were detected. Data were collected from two mice at each time point.

### Cell culture and pro-inflammatory cytokine stimulation

Human alveolar epithelial cells (A549 cell line) were purchased from RIKEN Cell Bank (Tsukuba, Japan) and maintained in Dulbecco’s Modified Eagle Medium (DMEM) supplemented with 10% (v/v) fetal bovine serum (FBS) at 37°C in 5% CO_2_. A 2-ml cell suspension (1 × 10^5^ cells/ml) was seeded into 6-well culture plates and incubated for 24 h to allow cell attachment. The cells were treated with human tumor necrosis factor α (TNFα PeproTech, Rocky Hill, NJ, USA), interleukin-1β (IL1β PeproTech), and lymphotoxin-α2/β1 (LTα2/β1, R&D Systems, Inc., Minneapolis, MN, USA), each at a concentration of 50 μg/ml, or PBS for negative control. Total RNA was extracted from these cells 24 h after cytokine stimulation.

### Quantitative real-time reverse transcription PCR

Total RNA was extracted using Sepasol-RNA I Super (Nacalai Tesque, Kyoto, Japan). Next, complementary DNA (cDNA) was synthesized using the iScript cDNA Synthesis Kit (Bio-Rad, Nazareth, Belgium). Then, quantitative real-time reverse-transcription PCR (RT-PCR) for human AID amplification was performed as described [5].

### Statistical analysis

Statistical analysis was performed using Stata 8.2 (StataCorp, College Station, TX, USA), except P value of Student’s *t* test for MALL size analysis, which was calculated using Prism 4.0 software (GraphPad Software, Inc., San Diego, CA, USA).

## Results

### Histological characteristics of the lungs in AIDon mice

In this study, we used mice with a single copy of AID-expressing transgene (AIDon mice). AIDon mice, in contrast to our previously reported AID transgenic mice driven by the same promoter but with multiple copy integration [10], did not suffer from early death from lethal T-cell lymphoma before the age of 1 year, and survived more than 90 weeks after birth. This trait, possibly due to lower expression of the AID transgene, greatly facilitated the observation of tumor development outside the lymphoid compartment.

**Fig 1:**
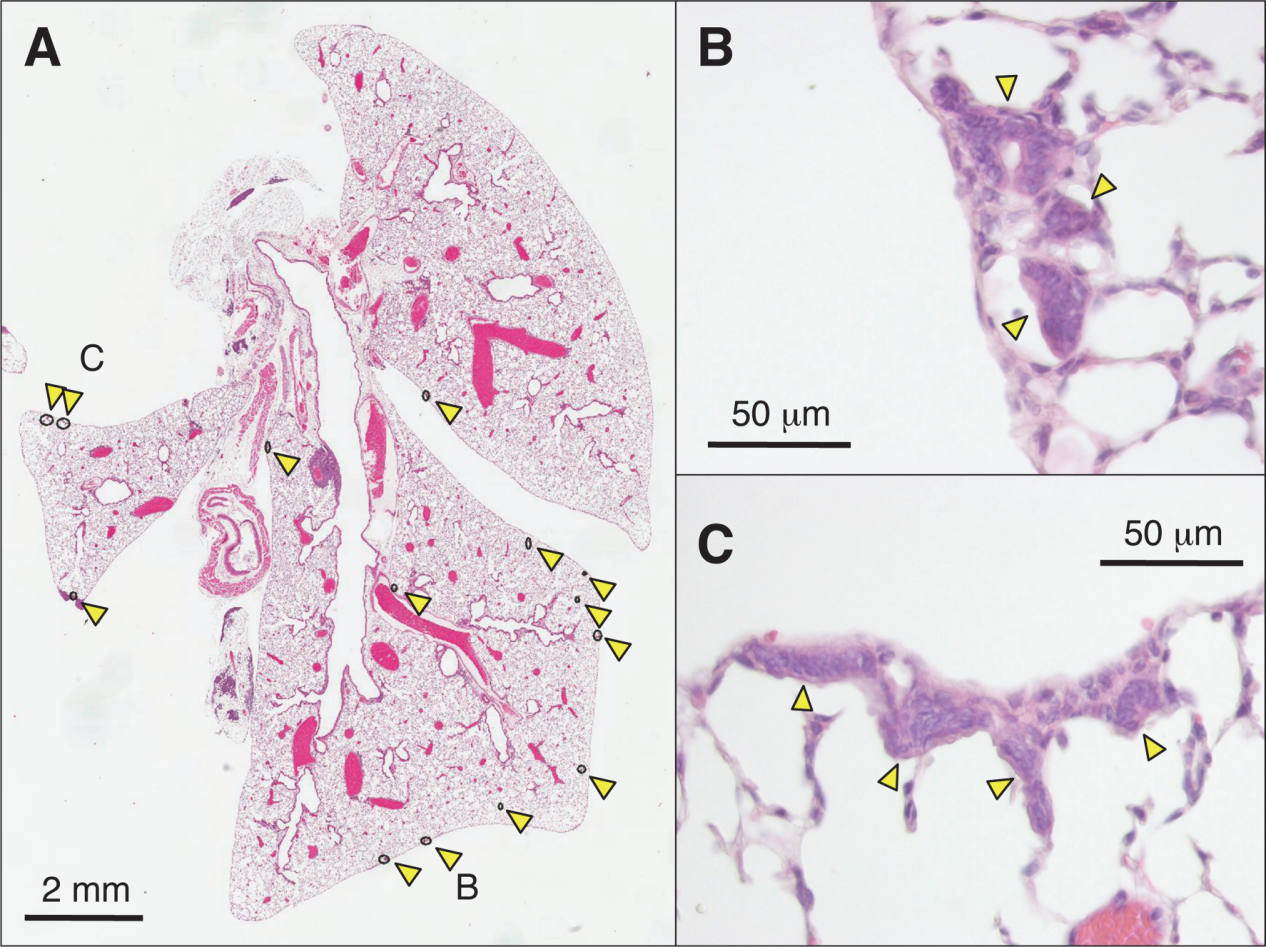
HE staining of the lung of an AID Tg mouse. A, An overview of the lung of an AID Tg mouse. Arrowheads and open circles indicate the locations of MALLs. B, C, Higher power views of a representative MALL in the lung of an AID Tg mouse. MALL is indicated by an arrowhead. HE, hematoxylin and eosin; AID, activation-induced cytidine deaminase; Tg, transgenic; MALL, mouse AAH-like lesion.

All AIDon mice older than 6 months developed multiple MALLs in the lung alveolar region as described previously [10] (Fig. 1). Each MALL, which consisted of 4–20 cuboidal or cylindrical cells in sections, was a patch of simple epithelium showing direct continuity with alveolar epithelium. MALL height was 6–60 μm and width was 12–160 μm. Elongated nuclei without atypia were located near the basement membrane (Fig. 1B). When MALL was sectioned by a plane parallel to the basement membrane, it appeared as cuboidal cells with round nuclei (Fig. 1C).

**Fig 2:**
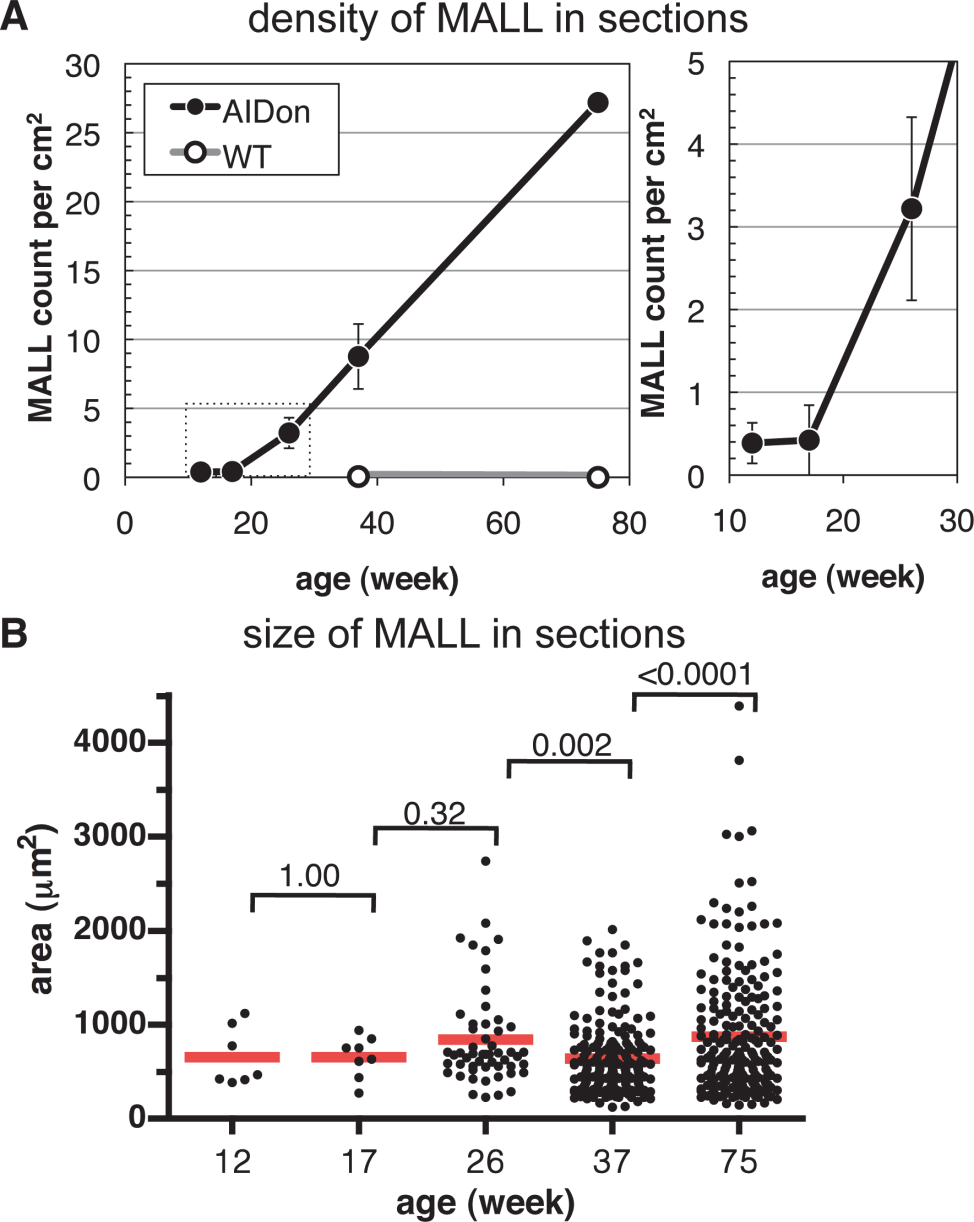
Time course of MALL count and size in the right lung of AIDon mice and wild-type mice. A, In the left panel, mean density of MALLs in sections per 1 cm^2^ is plotted against mice age in weeks. A solid line with closed symbol and a gray line with open symbol represent AIDon and wild-type mice, respectively. Error bars represent standard errors for data from three mice, except for a wild-type mouse and a 75-week-old AIDon mouse each of which was analyzed singly. Area of a dotted rectangle was enlarged in the right panel. B, Cross-sectional area with individual MALLs plotted. Mean values are indicated by red lines. P values calculated by t-test between groups are indicated by brackets.

MALL count was examined by averaging the density in 11 sections of the right lung at 100-μm intervals for mice between 12 and 75 weeks of age (Fig. 2A). MALL was barely detectable in 12- and 17-week-old mice, but was detected at 26 weeks. The number of MALLs increased with age. At 37 weeks, MALL density on sectioning was 4.3 ± 0.8/cm^2^ (mean ± standard error, n = 3), corresponding to an estimate of 860 MALLs in bilateral lungs (see Methods for the calculation). Among the 64 AIDon mice examined, only 7 (11%) developed macroscopically visible lung tumors (3 adenomas and 4 adenocarcinomas), which is comparable with lung tumor incidence in the original line of AID transgenic mice [11]. Considering the large number of MALLs in aged mouse lung, we speculate that most MALLs fail to grow as macroscopic tumors.

Varying sizes (based on cross-sectional area) of individual MALLs from mice of different ages are plotted in Fig. 2B. The mean size of a MALL appeared to be relatively constant over time. Although statistically significant differences were detected between 26 weeks and 37 weeks (P = 0.002), and between 37 weeks and 75 weeks (P < 0.0001), the mean value difference was small and did not seem to have biological significance. The occasional appearance of big MALLs in 75-week-old mice may indicate that a limited number of MALLs continue to grow, eventually becoming visible tumors on gross inspection.

### Lung cancer-related gene mutation analysis in MALLs

Human AAH reportedly harbors significantly elevated frequencies of mutations in three lung cancer-related genes: *Trp53*, *Kras*, and *Egfr* [17–23]. To examine whether MALL has mutations comparable to AAH, mutations were analyzed in hotspot regions of the three genes: *Trp53* exons 5–8, *Kras* exon 2, and *Egfr* exons 19–21. We microdissected 110 MALLs, from which DNA was purified individually and subjected to gene-specific PCR and direct sequencing. While four (10.5%) of 38 MALLs showed five missense mutations in the *Trp53* gene, no missense mutation of the *Kras* or *Egfr* gene was found. The mutation frequency of the *Trp53* gene was comparable with previously published data on human AAH in the lung [17,18], but *Kras* and *Egfr* mutations were much fewer than in human AAH [19–23] (Table 1). Four out of the five missense *Trp53* mutations are predicted to compromise the protein function, although it is unclear whether these deleterious mutations played some roles in the development of MALLs (S1A and B Fig.).

**Table 1:** Frequencies of lung cancer-related gene mutations in MALLs in comparison to published data for human AAH.

	*Trp53*/*TP53*	*Kras*/*KRAS*	*Egfr*/*EGFR*		
	Exon 5–8	Exon 2	Exon 19	Exon 20	Exon 21
MALL	10.5% (4/38)	0% (0/35)	0% (0/22)	0% (0/10)	0% (0/19)
AAH	0.0% Yamasaki, 2000 [17]	33.0% Sakamoto, 2007 [19]	35.0% Yoo, 2010 [23]		
	9.0% Slebos, 1998 [18]	26.7% Yoshida, 2005 [20]	25.0% Sakamoto, 2007 [19]		
		15.0% Cooper, 1997 [21]	3.0% Yoshida, 2005 [20]		
		39.0% Westra, 1996 [22]			

Numbers in parentheses indicate the number of MALLs with the gene mutation versus the number of MALLs examined.

Including intronic mutations, 14, 3 and 0 single-base substitutions were found in *Trp53*, *Kras* and *Egfr* gene, respectively. The substitution patterns of cytosine (C)-to-thymine (T) and guanine (G)-to-adenine (A) changes typical to cytidine deamination were not predominant in *Trp53* gene (14% [2/14], S1C Fig.). In both cases, mutated Cs were 5'-preceded by G, which was consistent with AID signature [24]. As shown in S1D Fig., 2 out of 3 substitutions seen in *Kras* gene were C-to-T change in a dinucleotide context of C 5'-preceded by T, which is a preferential target of APOBEC3 deamination [25]. However, small number of observed mutations in MALLs precludes inference of mutational process.

### Immunohistochemistry for airway epithelial cell markers

**Fig 3:**
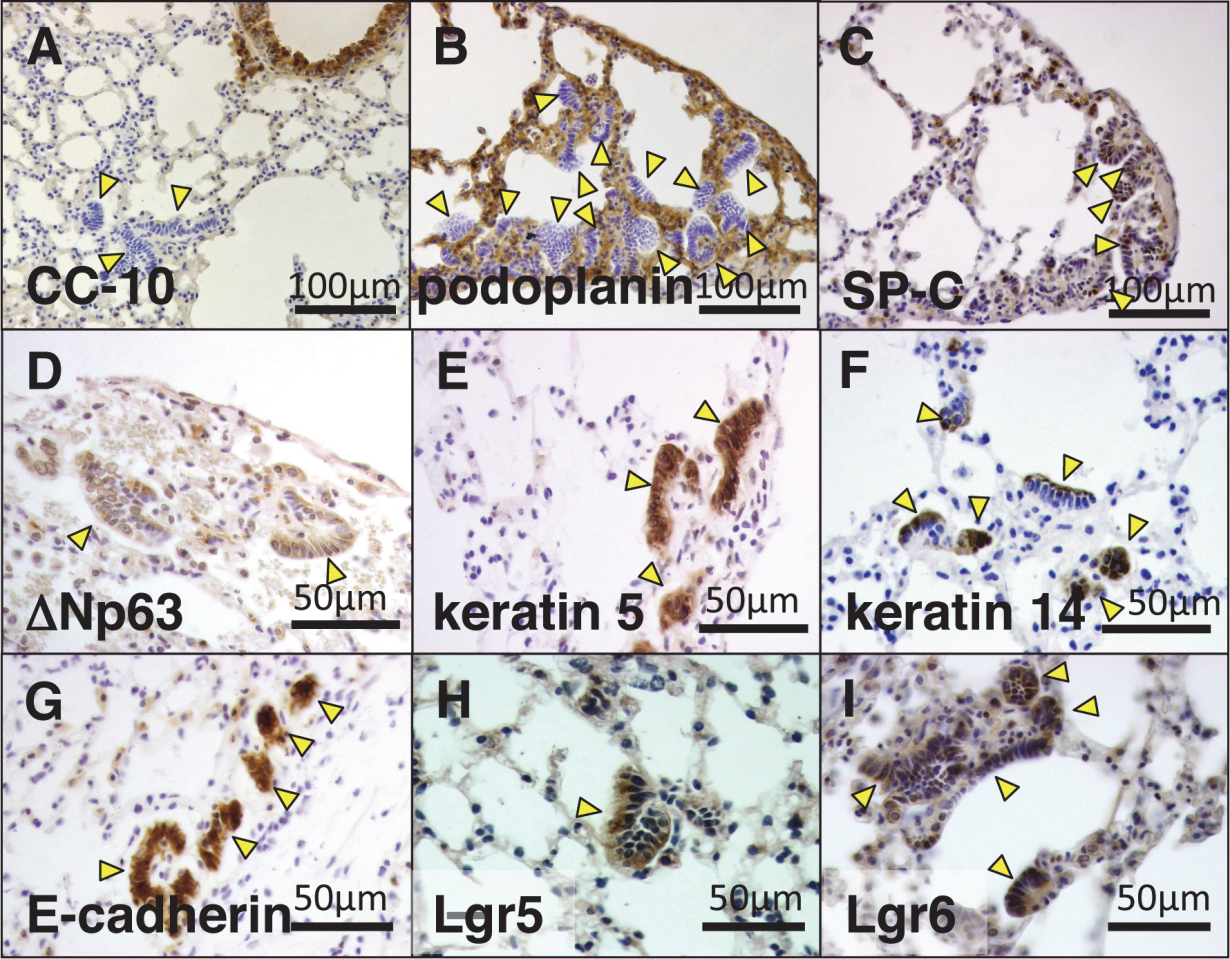
Immunohistochemistry of MALL for airway epithelial cell markers and lung regeneration markers. A, CC-10, clara cell marker and B, podoplanin, alveolar type I cell marker were negative. C, SP-C, alveolar type II cell marker was partially positive in MALL cells. D, ΔN p63; E, keratin 5; F, keratin14; G, E-cadherin; H, Lgr5; and I, Lgr6 were all positive in MALLs. Brown signal indicates positive staining by diaminobenzidine and blue signal indicates nuclear counter staining by hematoxylin. MALL is indicated by an arrowhead. CC-10, Clara cell 10kd protein; SP-C, surfactant protein C; E-cadherin, epithelial cadherin; Lgr, leucine-rich repeat containing G protein-coupled receptors.

To characterize the differentiation status of MALL, we performed immunohistochemistry for three markers specific to three kinds of distal airway epithelial cells: podoplanin in alveolar type-1 (AT1) cells, SP-C in alveolar type-2 (AT2) cells, and CC-10 in Clara cells. MALLs were partially positive for SP-C, but negative for CC-10 and podoplanin (Fig. 3A–C). This finding suggests that AT2 cells may be present in MALL.

The dormant nature of MALL and the paucity of mutations in lung cancer-related genes prompted us to speculate that MALL might be a regenerative lesion rather than an early neoplasm. Recently, regenerative markers of distal airway cells were reported. In a mouse model displaying lung regeneration after H1N1 influenza virus infection, p63, keratin 5, and keratin 14 are expressed in regenerating alveolar cells [26]. In another study using human lung-derived cells, cells expressing E-cadherin, Lgr5, and Lgr6 possessed a stem cell-like capacity for self-renewal and bronchioalveolar differentiation [27]. Immunohistochemistry also revealed that MALL expressed ΔNp63, keratin 5, keratin 14, E-cadherin, Lgr5, and Lgr6 (Fig. 3D–I), thereby indicating that MALL retains a signature of alveolar regenerative tissue.

### Electron microscopic findings

**Fig 4:**
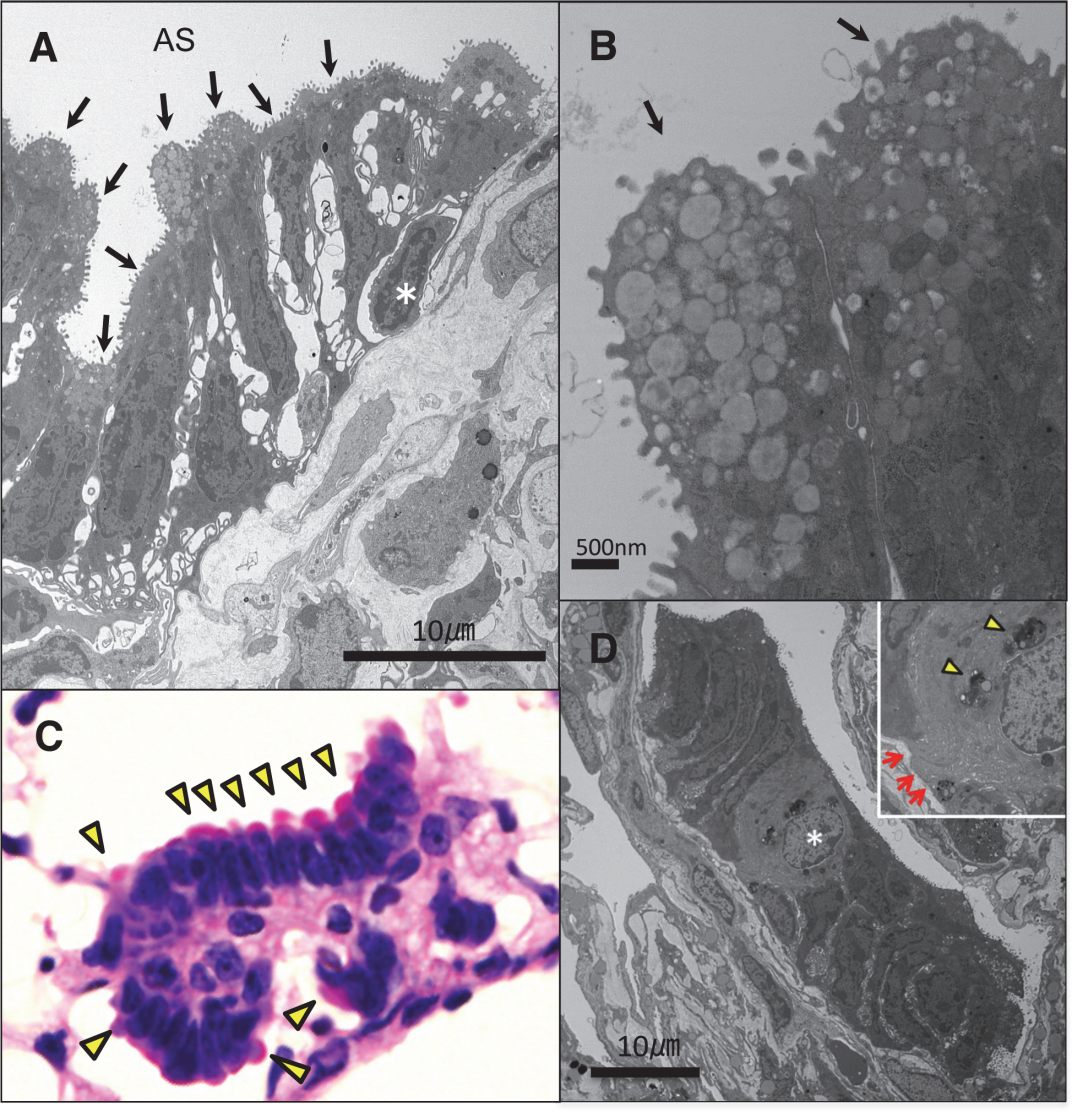
Characteristics of MALL cells as immature mucous cells. Electron microscopic findings of MALL: A, Overview of MALL cells. B, Magnified view of the apical part of MALL cells. Arrows indicate immature secretory vesicles in the cytoplasm of MALL cells. An asterisk indicates a basal cell adjacent to MALL cells. C, PAS staining demonstrates that positively stained materials are in the apex of MALL cells (arrowheads). D, Electron micrograph showing a phagocytic epithelial cell within MALL (asterisk). Inset, higher power view of the phagocytic epithelial cell. Arrows indicate the basement membrane. Arrowheads indicate residual bodies within the cell. AS, alveolar space; PAS, periodic-acid Schiff.

To further examine MALL characteristics, we assessed the lungs of AIDon mice via transmission electron microscopy. MALL consisted of a monolayer of cells on a basement membrane. The cells were columnar with elongated nuclei (Fig. 4A). Interestingly, all cells contained immature secretory vesicles in the apical portion of the cytoplasm without lamellar bodies (Fig. 4B). These vesicles were positive for PAS staining, indicating the presence of mucin (Fig. 4C). These findings indicate that MALL cells are, in fact, immature mucous cells. As human AAH contains lamellar bodies [28], MALL structure is distinct from AAH, initial nomenclature notwithstanding.

In addition, we detected basal cell-like cells, which are not normally seen in the distal lung of the mouse [29], on the basal side of MALL cells (Fig. 4A). Strong expression of keratins 5 and 14 suggests that MALL originated from this basal cell-like cell. Furthermore, we occasionally found phagocytic cells within MALLs (Fig. 4D). These cells had direct contact with the basement membrane and residual bodies in the cytoplasm. With this observation, we concluded that these cells were actually phagocytic epithelial cells. These cells represent a physiological response to the apoptosis of their epithelial neighbors [30,31]. Therefore, this finding suggests that clearance of dead tissue by epithelial cells occurs in the distal airway epithelium in AIDon mice.

### Enhanced cell death in the tissue of AIDon mice

**Fig 5:**
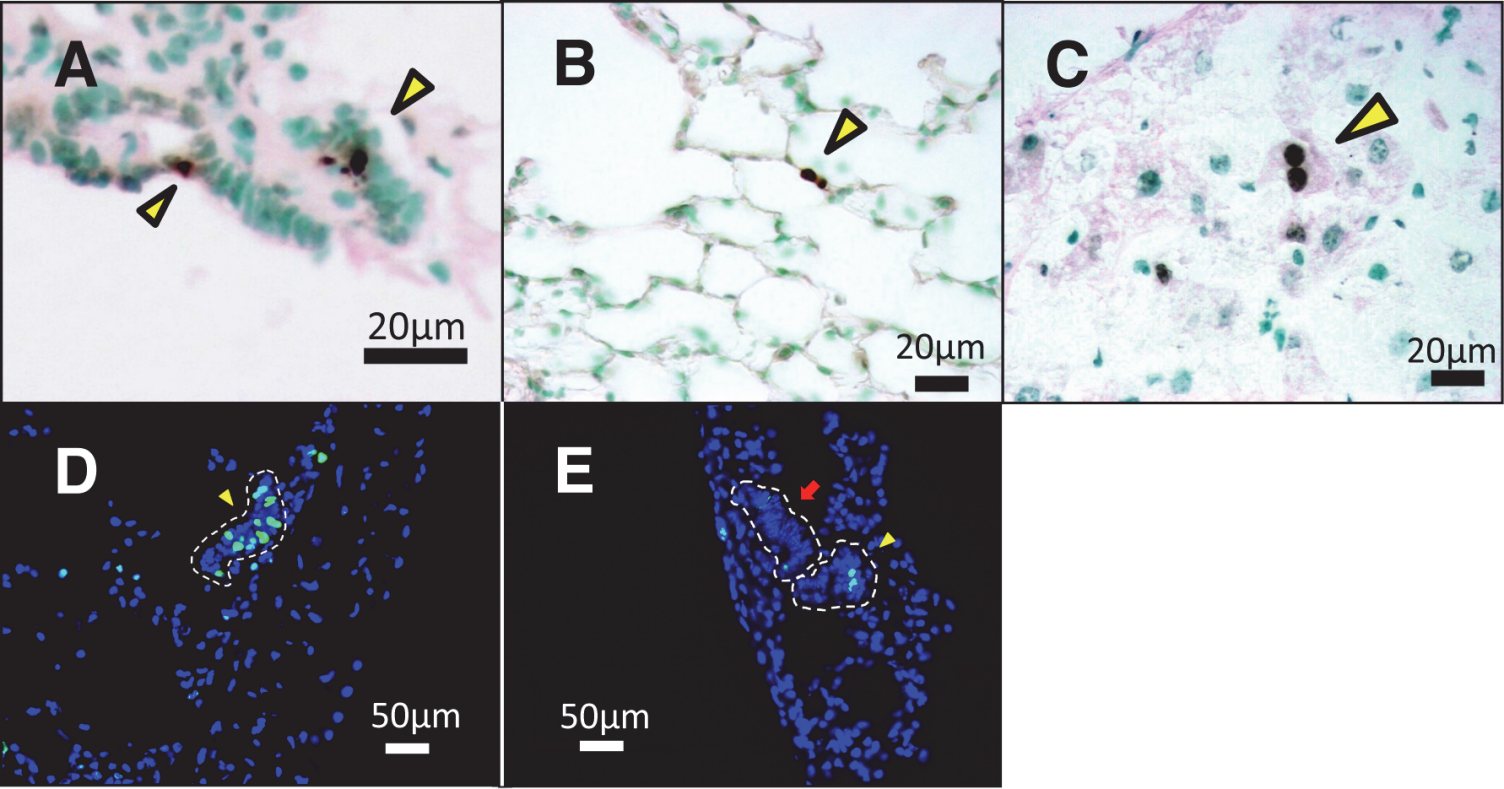
Cell deaths and cell proliferation in AID transgenic mice. TUNEL staining of A, MALL; B, Lung of AID Tg mouse; and C, Liver of AID Tg mouse. Arrowheads indicate TUNEL positive cells. Cell proliferation assay by EdU labeling in the lung of AID Tg mice and WT mice: D, Representative figure of EdU-positive MALL on day 1. An arrowhead indicates EdU-positive MALL (the region is surrounded by a dashed line). E, Representative figure of the lung of AID Tg mice on day 20. An arrowhead indicates EdU-positive MALL and an arrow indicates EdU-negative MALL. The region of each MALL is surrounded by a dashed line. TUNEL, terminal deoxynucleotidyl transferase (TdT)-mediated dUTP nick endlabeling; EdU, 5-ethynyl-2’-deoxyuridine.

The presence of phagocytic epithelial cells in MALLs prompted us to speculate that lung cell death occurs more frequently in AIDon mice than WT mice. In the TUNEL assay, we occasionally found dead cells within MALLs (Fig. 5A). However, dead cells were recognized even in the area outside MALL. The occurrence of dead cells outside MALL in AIDon mice lungs (0.43%) was significantly higher than in WT mice (0.09%, P = 0.013; Fig. 5B, Table 2). To examine whether AID cytotoxicity is specific to the lung, we performed TUNEL staining of liver tissue. The number of TUNEL-positive hepatocytes increased in AIDon mice compared with WT mice (6.0% vs. 4.8%, P = 0.036; Fig. 5C, Table 2). This finding clearly indicates that AID-induced cell death is not specific to the lung.

**Table 2:** TUNEL-positive and negative cells in lung alveolar wall cells and hepatocytes of AID Tg and WT mice.

TUNEL				
		Positive	Negative	P
Lung	AID Tg	14 (0.43%)	3215	.013
	WT	3 (0.09%)	3188	
Liver	AID Tg	186 (6.0%)	2910	.036
	WT	144 (4.8%)	2877	

Numbers in parentheses indicate the ratio of TUNEL positive cells to TUNEL negative cells.

### Cell proliferation in MALLs

MALL consists of multiple immature mucous cells and a limited number of basal cells morphologically similar to those of bronchi, which serve as stem cells for bronchial epithelia. If immature mucous cells are derived from these basal cells, it can be hypothesized that the generation of MALL should accompany cell division. To verify this hypothesis, we carried out a proliferation assay by 5-ethynyl-2'-deoxyuridine (EdU) incorporated into replicating DNA. First, we administered once-daily intraperitoneal injections of EdU to AIDon mice for 7 consecutive days. Half of the mice were sacrificed for lung inspection one day after the last EdU injection (day 1). We found a few EdU-positive cells within MALLs, indicating that these lesions have the potential to carry proliferating cells. After quantitative analysis of acquired images, 91.1% (41/45) of MALLs were found to contain EdU positive cells (Fig. 5D).

Next, we examined the lungs of the remaining half of AIDon mice on day 20. Interestingly, only 44.2% (19/43) of MALLs had EdU positive cells (P < 0.01 by chi-square test, Fig. 5E). This decrease in EdU positivity during the first 19 days can be interpreted by a scenario whereby established MALLs turn into normal alveolar tissue as new, unlabeled MALLs are generated. This transformation is believed to lower EdU positivity. Another scenario is that MALL cells undergo cell death and are eliminated. These two possibilities are not mutually exclusive. Which outcome is dominant is unclear at present.

Judging from the gradual increase in the number of MALLs (Fig. 2A), it seems likely that the rate of MALL generation slightly exceeds the disappearance rate.

### MALL and lung tumor

**Fig 6:**
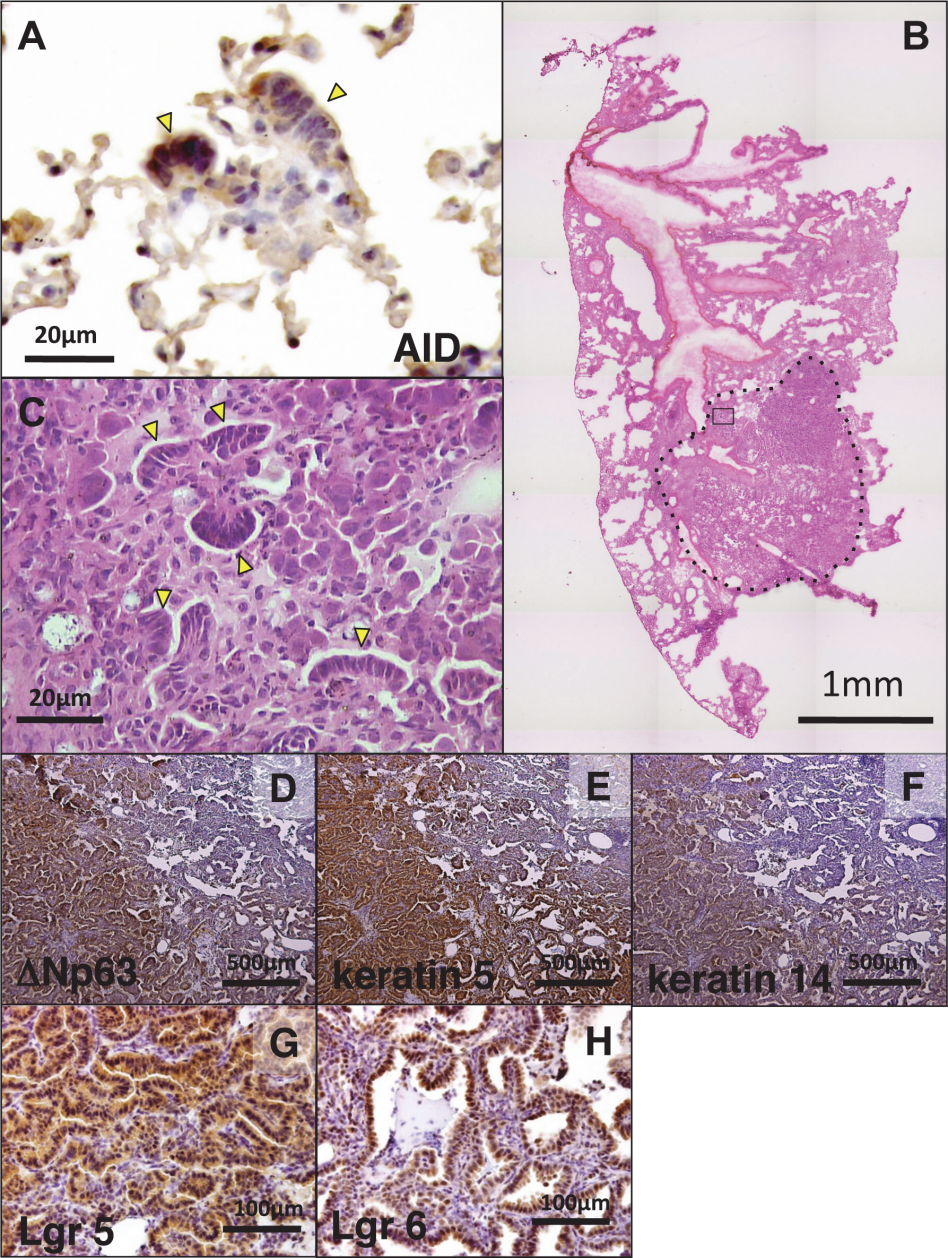
Evidences suggesting the link between AID and lung cancer. A, Immunohistochemistry for AID in MALLs. MALL is indicated by an arrowhead. B, HE staining of the lung of AID Tg mice with lung adenoma. An adenoma is demarcated by a dotted line. Region indicated by a rectangle is enlarged in C. C, Arrowheads indicate MALLs within lung adenoma. Immunohistochemistry for D, ΔNp63: E, keratin 5; F, keratin 14; G, Lgr 5; and H, Lgr 6 in lung adenoma.

Our data suggest that MALL is not a tumor, but a proliferating mucous cell metaplasia with restricted longevity. To investigate the relationship between MALL and lung tumor, which develops in about 10% of AIDon mice, we first confirmed AID expression in MALL by immunohistochemistry (Fig. 6A). This result was consistent with a quantitative RT-PCR result, showing 3-fold higher AID expression in MALL than surrounding alveolar tissue (S2 Fig.). Next, we examined sections of adenoma from AIDon mice lung. Interestingly, we found that MALLs are occasionally embedded within tumorous tissue (Fig. 6B and C). Furthermore, most of the tumor cells expressed the lung regeneration markers p63, keratin 5/14, and Lgr 5/6 (Fig. 6, D–H). Expression of these regeneration markers was often limited to a subpopulation of tumor cells, probably reflecting an instance of intratumoral heterogeneity. The presence of MALL within a cancerous tumor and its proliferative potential suggest that lung tumor may have originated from MALL in AID transgenic mice.

### Induction of AID expression in human alveolar epithelial cells

**Fig 7:**
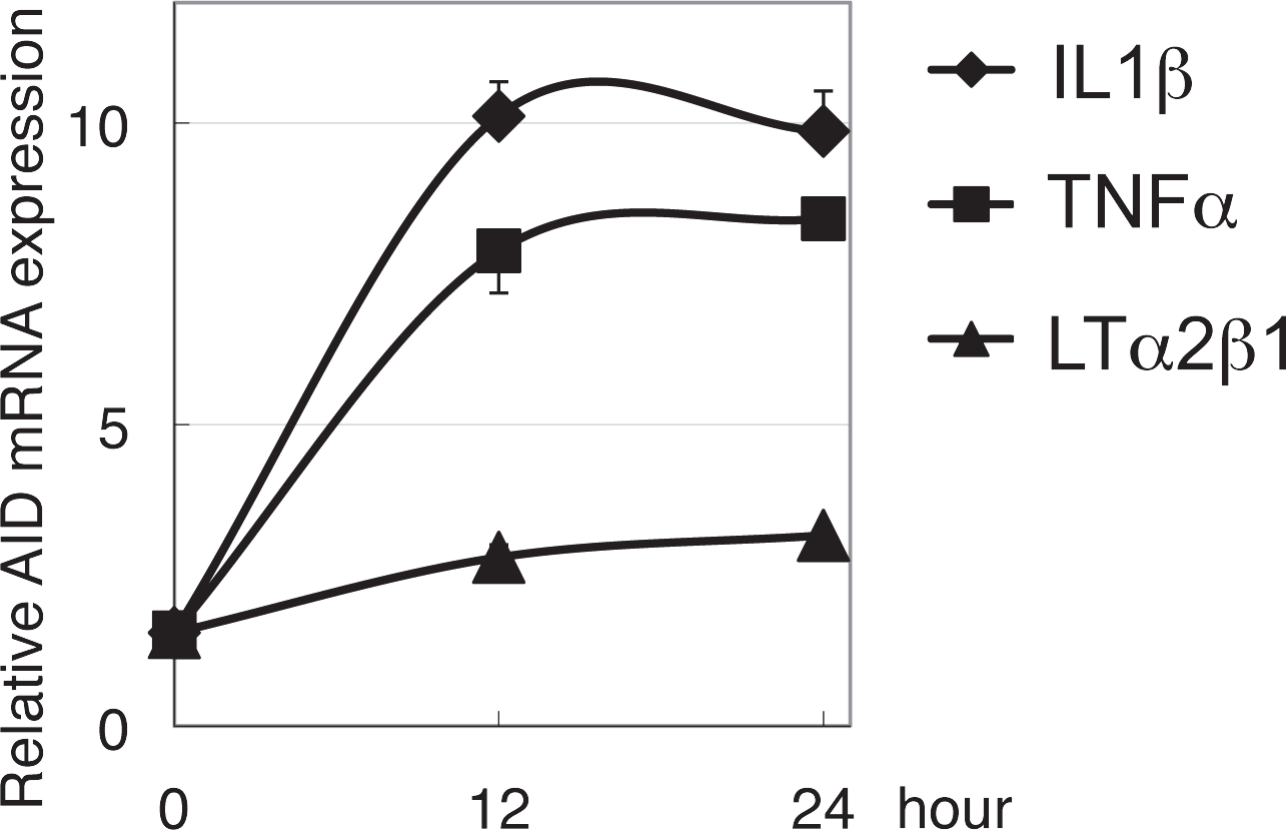
AID expression induced in human lung alveolar epithelial cell in response to pro-inflammatory cytokine stimuli. The plot shows the result of quantitative real-time reverse transcription PCR for AID mRNA from A549 human lung adenocarcinoma-derived cells stimulated with tumor necrosis factor (TNF) α, interleukin (IL) 1β or lymphotoxin (LT) α2/β1 for 12 and 24 h. Data are mean ± SE; n = 3. SE, standard error.

If AID expression is induced in alveolar epithelial cells by pro-inflammatory cytokines, as was shown for human cancers of the gastrointestinal tract [5–7], the possibility of AID involvement in human lung cancer would emerge. To examine whether inflammatory stimuli enhance AID expression in alveolar epithelial cells, the process was analyzed by quantitative RT-PCR in human lung adenocarcinoma-derived A549 cells. A549 cells were stimulated with TNFα, IL1β or LT α2/β1 for 12 and 24 h. RNA was then analyzed for AID mRNA expression by quantitative RT-PCR. AID expression was successfully induced 12 h after adding TNFα or IL1β, whereas induction by LTα2/β1 was marginal (Fig. 7).

## Discussion

In the present study, we proposed that MALL is a mucous cell metaplasia that develops after AID-induced lung alveolar injury and could be a precancerous lesion for lung cancer. In addition, we demonstrated that AID expression is induced in human alveolar cells by pro-inflammatory cytokines, suggesting the involvement of AID in human lung carcinogenesis. We started this study from an assumption that MALL may be a very early stage tumor much like human AAH. However, mutational analysis and electron microscopic analysis both indicated clear differences between MALL and AAH. The expression of recently identified lung regeneration markers prompted us to rethink the role of MALL in tumorigenesis.

The matter of AID cytotoxicity was previously reported [32]. However, such cytotoxicity was noticed only when the DNA repair machinery involved in homologous recombination is suppressed. Olivier et al directly proved the existence of genome-wide mutations by AID in cultured mouse embryonic fibroblasts (MEFs) [33]. In their study, the number of nonsynonymous AID mutations were just 1.5 times more than the spontaneous mutations that occurred (3.8 versus 2.5 mutations per 1 megabase, calculated using the values in Supplementary Dataset Three in [33]). This indicates that AID genotoxicity is very mild. Moreover, Olivier et al reported that MEFs from AID transgenic mice harbored mutations in many histone genes, which are essential for cell survival.

We demonstrated increased cell death in the lungs and liver of AID transgenic mice using TUNEL assay (Fig. 5, Table 2). This is the first demonstration of AID cytotoxicity in animal tissue. The subtle but statistically significant increase in cell death was reasonable in light of AID’s mild toxicity. We envisage that low-frequency cell death occurs in other organs of AID transgenic mice. Whether tumor frequency is correlated with cell-death frequency remains to be investigated.

**Fig 8:**
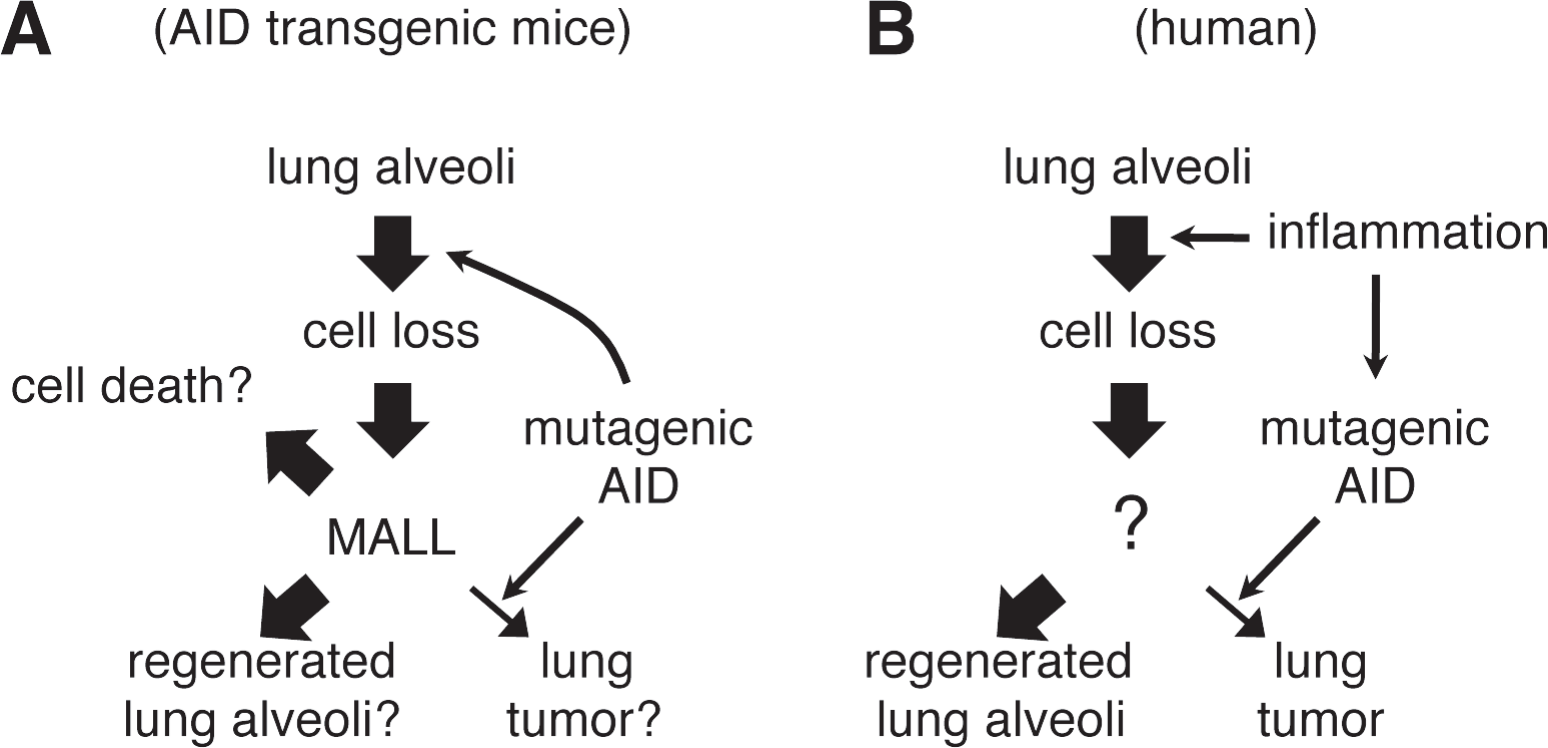
Models for the involvement of AID with carcinogenesis in murine and human lung. A, In alveoli of AID transgenic mice, mutagenic activity leads to stochastic cell death as a consequence of compromised function of some essential genes. This cell loss triggers MALL (mucous cell metaplasia). Expression of AID in MALLs may cause oncogenic mutations, stochastically giving rise to lung tumor. Most MALLs never contribute to tumor formation but resolve as normal alveolar tissue or disappear after cell death. B, In human lung, chronic inflammation causes alveolar cell damage. Sustained AID expression in yet uncharacterized regenerative tissue under chronic inflammation drives lung tumor formation.

Fig. 8A shows the current model of MALL in AID transgenic mice. In this mice line, AID is expressed from ubiquitous CAG promoter-driven transgenes from all over the body, including lung alveoli. As AID is a mutagenic enzyme, it exerts mild cytotoxicity and causes sporadic cell loss in the alveoli, which may trigger mucous cell metaplasia and MALL. The fate of MALL is not formally defined in this study. Further studies are necessary to determine the future of MALL, particularly regarding the mechanisms attending alveolar regeneration and cell death.

In our transgenic model, AID acts again to initiate cancer formation, probably from MALL. Because AID is expressed in MALL (Fig. 6A), some cells may eventually acquire oncogenic mutations. Mutations in tissue stem cells, in contrast to those occurring in differentiated cells with a limited lifespan, would have a greater potential for tumor formation. In this sense, basal cell-like cells in MALL may be the most relevant target of the AID-induced mutations leading to cancer. However, according to a recent report demonstrating dedifferentiation of committed tracheal epithelial cells into stem cells [34], immature mucous cells in MALL may also contribute to tumor formation if oncogenic mutations are introduced. Conversely, basal cell-like stem cells in MALL might be dedifferentiated from nascent but committed alveolar epithelia with oncogenic mutations already acquired via AID. Such dedifferentiation has been reported in other organs such as intestinal epithelia [35] and testicular germ cells [36].

If MALL is a regenerative lesion, why does it not appear in wild-type mice? The first possibility is that they do appear but at a low frequency. In normal mouse lung, epithelial loss may be rare with MALL frequency below a level detectable in a single section of lung: in our analysis of 55 sections of the right lung from five wild-type mice, only one MALL was identified (S3 Fig.). Such a low frequency may reflect the fact that turnover of the alveolar epithelium in adult mice is relatively slow (4–5 weeks) compared with tracheal and bronchial epithelium (2–7 days) [37,38]. The other possibility is that MALL occurs only in the context of AID overexpression. As mentioned, AID causes genome-wide mutations, especially in histone genes. Such AID-induced mutations may perturb normal cellular differentiation. In humans, mucous cell metaplasia occurs in the airway from trachea to bronchioles in chronic inflammatory lung disease [39–41]. This disease process implies a common mechanism between MALL and mucous cell metaplasia in the human airway.

As reported in the literature, intrabronchial instillation of keratinocyte growth factor (KGF or FGF-7) in rats resulted in diffuse proliferation of SP-C-producing cells on day 2 that were morphologically very similar to MALL [42]. This structure disappears on day 7. In a mouse lung injury model of H1N1 influenza virus infection, cell clusters expressing p63 and basal keratins 5 and 14 appear as a transient event [26]. These keratin 5-positive cells can show alveolar differentiation in vitro, exhibiting spheres of mono-layered cells in an air–liquid interface (ALI) culture. We observed similar thin spheres in an ALI culture of minced lung fragment from AID transgenic mice, but not from wild-type mice (S4 Fig.). In a study using human lung, a minor population of cells expressing E-cadherin, Lgr5, and Lgr6 possessed the capacity to differentiate into bronchioalveolar epithelium under the mouse kidney capsule [27]. We speculate that a MALL expressing these documented distal airway stem cell markers contains similar stem cells with the potential for alveolar differentiation.

The involvement of AID in human lung carcinogenesis is unclear. AID induces genome-wide mutations not only in B cells but also in epithelial cells [10,43]. Recently acquired genome sequence data on lung adenocarcinoma indicate that mutational signatures differ between high- and low-mutational burden groups. In the high-mutational burden group (more than 15 mutations per megabase), the signatures associated with smoking (mainly cytosine-to-adenine change) prevailed, whereas in the low-mutational burden group (less than 2.5 mutations per megabase), signatures associated with APOBEC family cytidine deaminases including AID (mainly C-to-T change) predominated [44] (see Supporting Figure Forty-four of ref. [44]). It is reported that 35% of human lung cancer expresses AID [12]. In the present study, we demonstrated that human alveolar epithelial cell line A549 expresses AID when stimulated with TNFα or IL1β (Fig. 7). Therefore, we envisage the model theorized below and depicted in Fig. 8B. In the presence of chronic inflammation, cells are frequently lost and replenished by a process of human lung regeneration that is currently unknown. Sustained expression of AID in human alveolar cells undergoing regeneration in an inflammatory milieu may induce oncogenic mutations, giving rise to lung cancer.

In conclusion, we demonstrated that MALL is a source of mucous cell metaplasia that appears transiently in the lung alveolar region after AID-induced lung injury. Tissue damage and tumor formation in AID transgenic mice may be triggered by genotoxic and/or mutagenic mechanisms peculiar to activation-induced cytidine deaminase. Finally, we emphasize the uniqueness of AID transgenic mice, whereby mild cell death occurs in the lung, liver, and probably other organs. This model will be useful for future studies of tissue regeneration and cancer development.

## Supporting Information

S1 Fig *Trp53* and *Kras* mutations in MALL. A. Structure of *Trp53* gene and sequenced region (red line) are shown. Rectangles and numbers indicate exons, from the 5th to the 8th of which encodes DNA binding domain. Position of the initiation codon is indicated by an arrow labeled with ‘ATG’. Protein-coding exons are indicated by filled rectangles. Pale blue boxes indicates 5'- or 3'-untranslated (UT) regions. B. List of *Trp53* mutations observed by direct sequencing. Position (nucleotide distance from the initiation codon), location in relation to exon/intron structure, patterns of mutations, and functional prediction according to IARC TP53 database (http://p53.iarc.fr) are shown. C. Base substitution pattern seen in *Trp53* gene of MALLs. Percentages in parentheses are compositions of the indicated bases in the sequenced region. D. Base substitution pattern seen in *Kras* gene of MALLs.
(TIF)

S2 Fig AID mRNA expression in MALL. AID mRNA expression of MALL and surrounding alveolar tissues, which were microdissected from HOPE-fixed lung of a 74-week-old AIDon mouse, were analyzed by quantitative RT-PCR. One hundred thirteen MALLs and alveolar tissues each with a total area of 0.5 mm^2^ were subjected to RNA purification. Standard curve was plotted using serially diluted cDNA from liver tissue of AIDon mouse as PCR templates. Calculated values were normalized by the level of hypoxanthine phosphoribosyltransferase mRNA. The values are represented with that of AIDon liver as 1. Error bar represents standard error of triplicate measurements. Method of intron-spanning quantitative PCR specific to CAG promoter–driven AID transgene was described [15].
(TIF)

S3 Fig HE staining of the lung of wild-type mouse. MALLs are very rare in wild-type mice. A, Out of 55 4 μm-thick sections from 37- and 75-week-old wild-type mice, only one MALL was found in a section of 37-week-old mouse lung. B, Region of dotted rectangle is enlarged. MALL is indicated by an arrowhead. Scale bar is 200 μm in A and 50 μm in B.
(TIF)

S4 Fig Comparison of ALI culture of the lung from wild-type mouse and AIDon mouse. The lung tissues from 82-week-old wild-type (A, B, and C and AIDon mice (D, E, and F) were minced and cultured via the air-liquid interface culture (ALI) method using collagen gel with Ham's F-12 medium supplemented with 20% fetal bovine serum and 50 μg/ml gentamicin according to Ootani et al. (Nat. Med. vol. 5, p. 701–706, 2009), A, B, D, E, Stereomicroscopic images of live culture. C, F, Sectional image of formalin-fixed paraffin embedded sample after HE staining. Scale bar is 500 μm. Thin spheres were formed from AIDon mice, but not WT mice.
(TIF)
