# Correction: Chronic Lung Injury by Constitutive Expression of Activation-Induced Cytidine Deaminase Leads to Focal Mucous Cell Metaplasia and Cancer

**DOI:** 10.1371/journal.pone.0136807

**Published:** 2015-08-21

**Authors:** Jiro Kitamura, Munehiro Uemura, Mafumi Kurozumi, Makoto Sonobe, Toshiaki Manabe, Hiroshi Hiai, Hiroshi Date, Kazuo Kinoshita

There are two errors in the Materials and Methods section and the Results section.

The final sentence of the fourth paragraph in the Materials and Methods section is incorrect. The correct sentence is: Using the following values (N = 4.3, D = 0.005, bilateral lung volume of 1.0 cm3) for a 37-week-old mouse, the total number of MALL in the lungs is calculated as N/D × lung volume = 8.8/0.005 × 1.0 = 1760.

The fourth sentence of the third paragraph of the Results section is incorrect. The correct sentence is: At 37 weeks, MALL density on sectioning was 8.8 ± 2.4/cm2 (mean ± standard error, n = 3), corresponding to an estimate of 1760 MALLs in bilateral lungs (see Methods for the calculation).
