# Supplementary figures and images for: Chronic Lung Injury by Constitutive Expression of Activation-Induced Cytidine Deaminase Leads to Focal Mucous Cell Metaplasia and Cancer
Source: PLoS One. 2015 Feb 6;10(2):e0117986. doi: 10.1371/journal.pone.0117986 (PMC4320068; doi:10.1371/journal.pone.0117986)

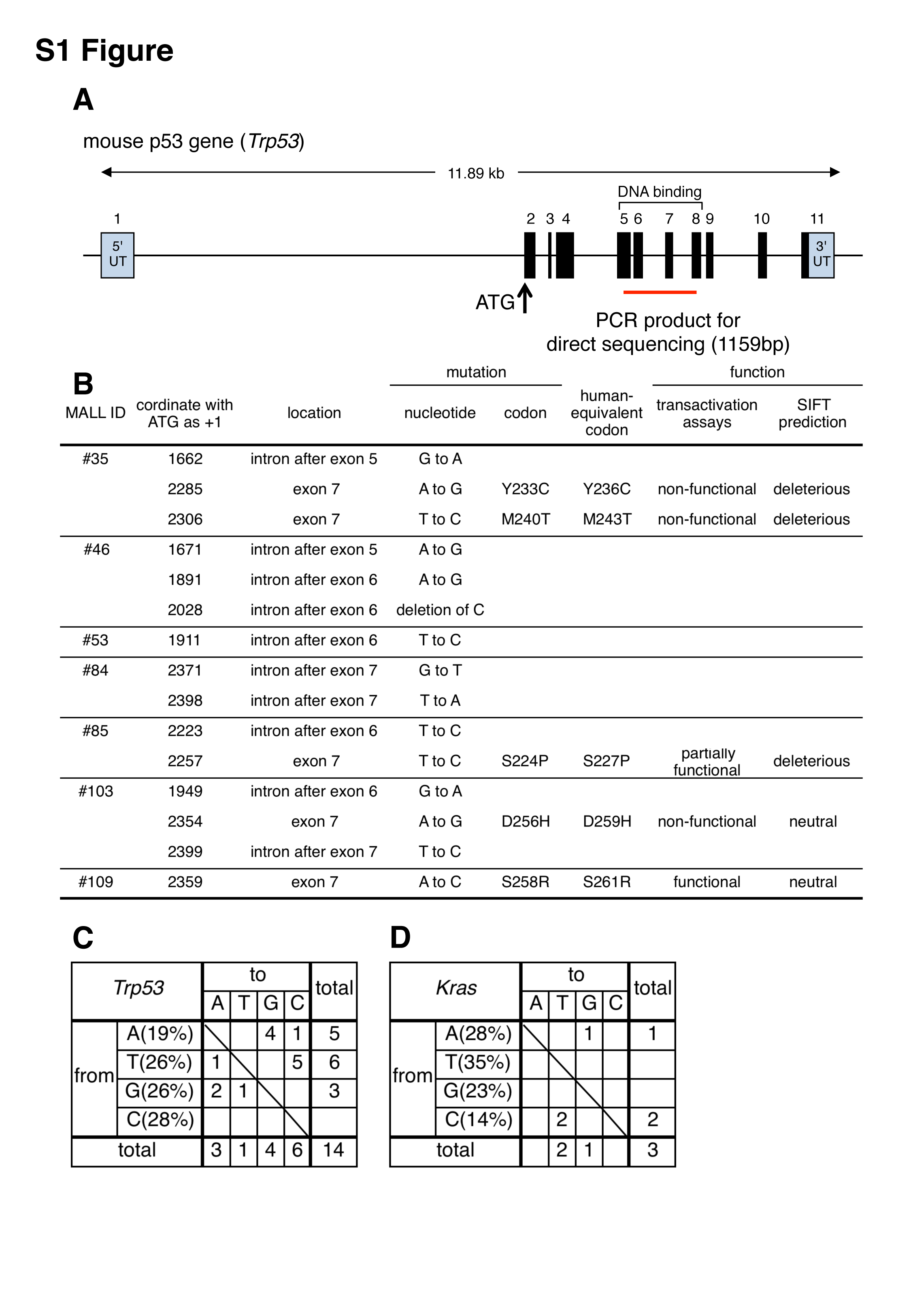

Supplement: S1 Fig — A. Structure of Trp53 gene and sequenced region (red line) are shown. Rectangles and numbers indicate exons, from the 5th to the 8th of which encodes DNA binding domain. Position of the initiation codon is indicated by an arrow labeled with ‘ATG’. Protein-coding exons are indicated by filled rectangles. Pale blue boxes indicates 5'- or 3'-untranslated (UT) regions. B. List of Trp53 mutations observed by direct sequencing. Position (nucleotide distance from the initiation codon), location in relation to exon/intron structure, patterns of mutations, and functional prediction according to IARC TP53 database (http://p53.iarc.fr) are shown. C. Base substitution pattern seen in Trp53 gene of MALLs. Percentages in parentheses are compositions of the indicated bases in the sequenced region. D. Base substitution pattern seen in Kras gene of MALLs. (TIF) [file pone.0117986.s001.tif]

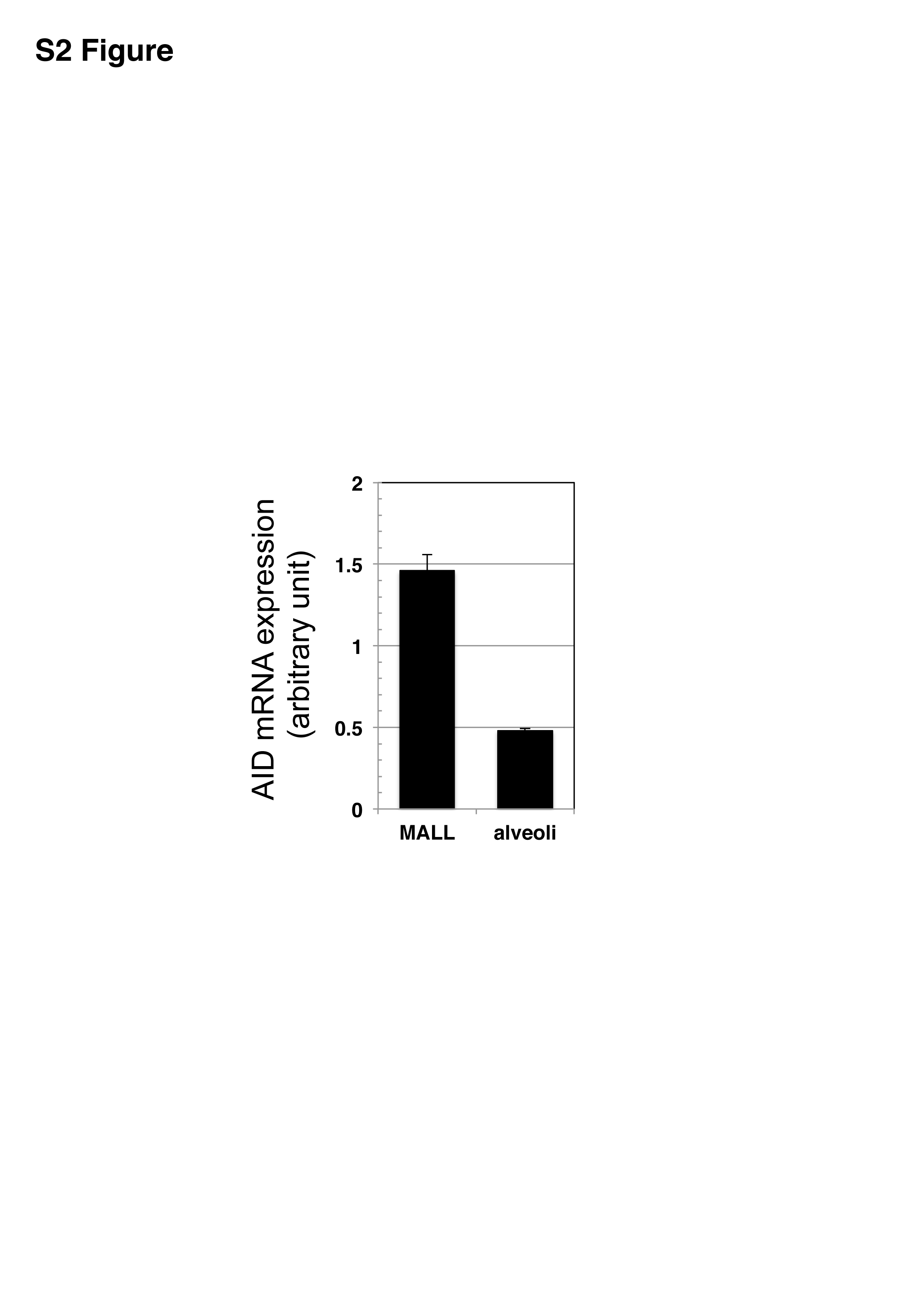

Supplement: S2 Fig — AID mRNA expression of MALL and surrounding alveolar tissues, which were microdissected from HOPE-fixed lung of a 74-week-old AIDon mouse, were analyzed by quantitative RT-PCR. One hundred thirteen MALLs and alveolar tissues each with a total area of 0.5 mm2 were subjected to RNA purification. Standard curve was plotted using serially diluted cDNA from liver tissue of AIDon mouse as PCR templates. Calculated values were normalized by the level of hypoxanthine phosphoribosyltransferase mRNA. The values are represented with that of AIDon liver as 1. Error bar represents standard error of triplicate measurements. Method of intron-spanning quantitative PCR specific to CAG promoter–driven AID transgene was described [15]. (TIF) [file pone.0117986.s002.tif]

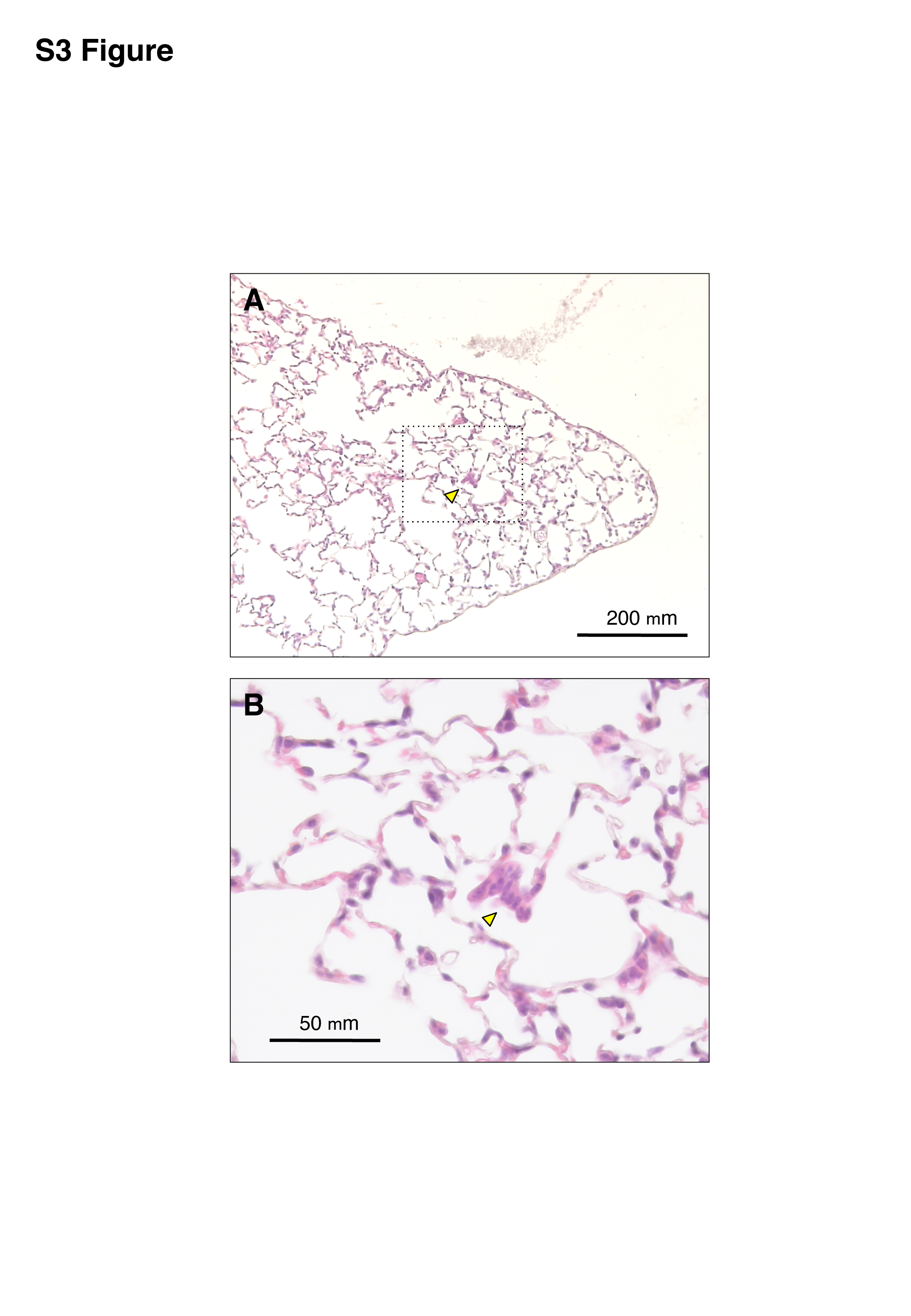

Supplement: S3 Fig — MALLs are very rare in wild-type mice. A, Out of 55 4 μm-thick sections from 37- and 75-week-old wild-type mice, only one MALL was found in a section of 37-week-old mouse lung. B, Region of dotted rectangle is enlarged. MALL is indicated by an arrowhead. Scale bar is 200 μm in A and 50 μm in B. (TIF) [file pone.0117986.s003.tif]

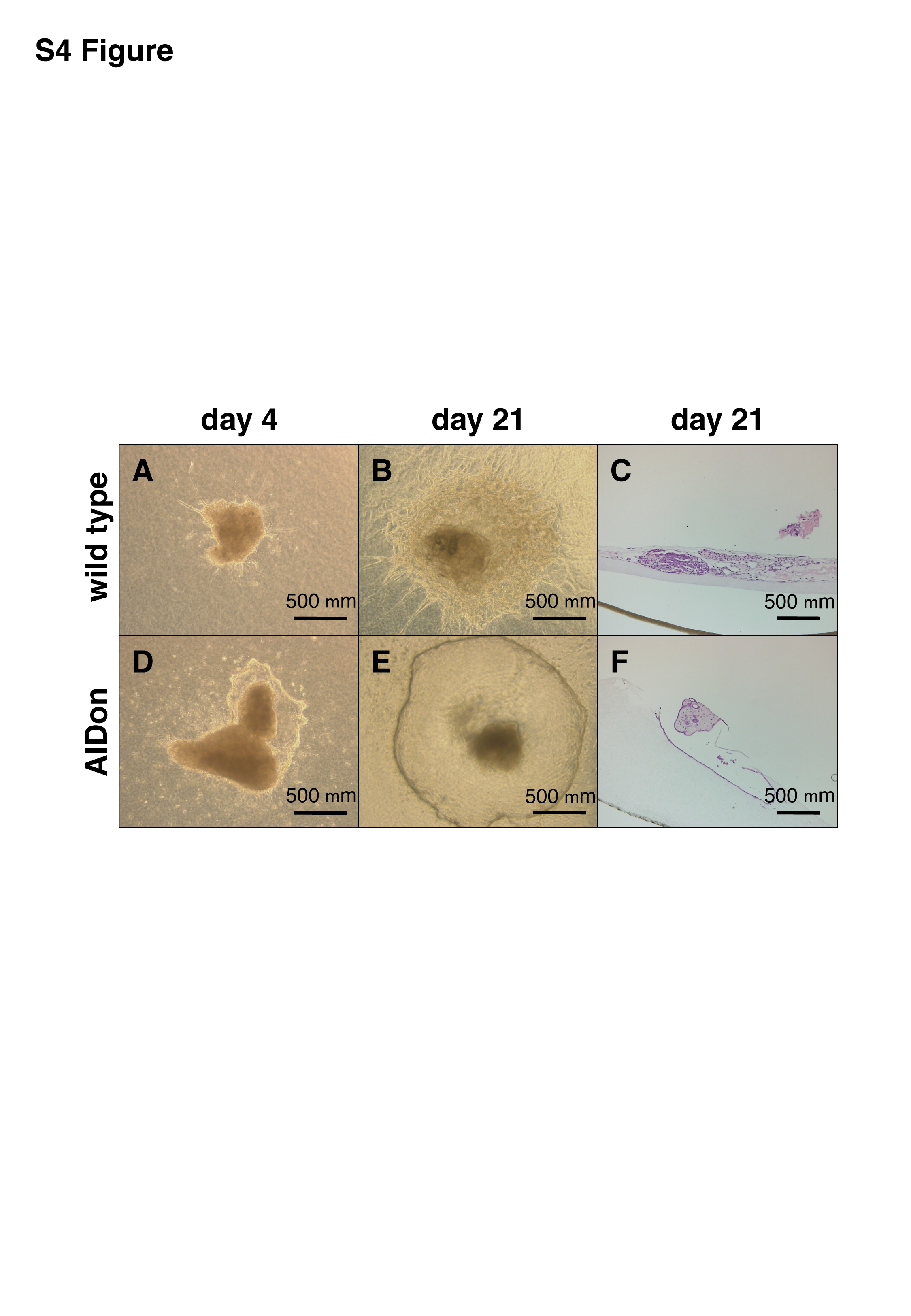

Supplement: S4 Fig — The lung tissues from 82-week-old wild-type (A, B, and C and AIDon mice (D, E, and F) were minced and cultured via the air-liquid interface culture (ALI) method using collagen gel with Ham's F-12 medium supplemented with 20% fetal bovine serum and 50 μg/ml gentamicin according to Ootani et al. (Nat. Med. vol. 5, p. 701–706, 2009), A, B, D, E, Stereomicroscopic images of live culture. C, F, Sectional image of formalin-fixed paraffin embedded sample after HE staining. Scale bar is 500 μm. Thin spheres were formed from AIDon mice, but not WT mice. (TIF) [file pone.0117986.s004.tif]
